# Geographic access to radiotherapy facilities in Japan

**DOI:** 10.1093/jrr/rrag004

**Published:** 2026-02-26

**Authors:** Yuhei Koike, Satoaki Nakamura, Hodaka Numasaki, Noboru Tanigawa

**Affiliations:** Department of Radiology, Kansai Medical University, 2-5-1 Shinmachi, Hirakata, Osaka, 573-1010, Japan; Division of Radiation Oncology, Kansai Medical University Hospital, 2-3-1 Shinmachi, Hirakata, Osaka, 573-1191, Japan; Department of Radiology, Kansai Medical University, 2-5-1 Shinmachi, Hirakata, Osaka, 573-1010, Japan; Division of Radiation Oncology, Kansai Medical University Hospital, 2-3-1 Shinmachi, Hirakata, Osaka, 573-1191, Japan; Department of Medical Physics and Engineering, Osaka University Graduate School of Medicine, 1-7 Yamadaoka, Suita, Osaka, 565-0871, Japan; Department of Radiology, Kansai Medical University, 2-5-1 Shinmachi, Hirakata, Osaka, 573-1010, Japan

**Keywords:** geographic access, radiotherapy, IMRT, brachytherapy, travel time

## Abstract

Daily attendance requirements for radiotherapy (RT) make geographic accessibility a critical determinant of treatment adherence and completion. Although facility surveys indicate that advanced modalities such as intensity-modulated radiotherapy (IMRT) and brachytherapy are concentrated in urban centers, precise nationwide quantification of the resulting patient travel burden remains lacking. This study provides the first nationwide, modality-stratified assessment of geographic access to RT in Japan. Using the Open Source Routing Machine and a high-resolution dataset of 176 964 household-weighted 1-km mesh centroids, we calculated driving times to the nearest external-beam radiotherapy (EBRT), IMRT and brachytherapy facilities. Beyond standard distribution metrics, we generated high-resolution ‘penalty maps’ to quantify the incremental time tax imposed by advanced modality requirements. Although EBRT access was uniformly short nationwide, with a median travel time of 6.48 min, this increased to 8.26 min for IMRT and 14.06 min for brachytherapy. Crucially, the proportion of the population facing poor access (≥120 min) doubled from 0.24% for EBRT to 0.48% for brachytherapy. The spatial analysis identified specific ‘newly poor-access’ areas—regions that are accessible for EBRT but become remote when advanced care is needed—forming coherent geographic clusters in mountainous and island zones. These findings demonstrate that modality requirements introduce meaningful inequities despite strong national EBRT infrastructure. These indicators provide a vital evidence base for spatially optimizing resources to mitigate travel burdens for Japan’s aging, mobility-limited population.

## INTRODUCTION

Radiotherapy (RT) is a core component of modern cancer treatment and typically requires daily attendance over several weeks. Consequently, geographic accessibility—measured by travel distance or time—plays a critical role in whether patients can initiate, complete and adhere to recommended treatment. In Japan, RT infrastructure exhibits marked regional variation in the availability of advanced modalities. Nationwide analyses using administrative and survey data have shown that, while linear accelerators are broadly distributed, intensity-modulated radiotherapy (IMRT) and brachytherapy remain concentrated in urban regions, with substantial inter-prefectural heterogeneity in adoption rates [1–8]. Recent national reports further indicate gaps in workforce distribution and treatment capacity, particularly in rural prefectures.

Much of the quantitative evidence on RT access, however, comes from studies conducted in other countries. In the USA, nationwide assessments have estimated straight-line distances from residential ZIP Code Tabulation Areas to RT facilities, revealing that 1.8% of the US population lives more than 50 miles from the nearest facility, with persistent gaps in rural regions [9]. Canada has extended this approach by modeling driving times using OpenStreetMap-based routing; 9.3% of the Canadian population resides more than 2 hours from the nearest RT facility, and 120 min has been formalized as a clinically meaningful threshold for ‘poor access’. This modeling framework has also been used to evaluate the impact of potential new RT centers on national travel burden, providing a basis for spatial optimization of RT services [10]. More recently, a global geospatial analysis quantified worldwide disparities in RT access and demonstrated how alternative facility placement scenarios could reduce travel-time burdens [11].

Complementing these population-level assessments, patient-level studies have shown that travel burden has concrete clinical consequences. Narrative reviews and observational analyses have demonstrated that greater travel distance is associated with delays in cancer diagnosis, treatment selection and treatment completion [12]. In breast cancer care, longer travel distance has been associated with higher mastectomy rates and lower use of adjuvant RT [13], whereas findings in prostate cancer have been more mixed, with some studies reporting limited associations between travel time and treatment choice [14]. A recent systematic review further synthesized heterogeneous evidence, with some studies linking increased travel distance or time to poorer survival, reduced adherence or lower likelihood of receiving guideline-concordant treatment, while others found no clear association across multiple malignancies [15]. Evidence from Japan similarly highlights the clinical relevance of geographic barriers: national registry-based studies in pediatric and breast cancer care have shown that patients in rural regions face longer travel requirements and reduced access to specialized services [16, 17].

Despite this growing international and domestic evidence, important gaps remain. First, no nationwide, time-based analysis of RT access has been conducted for Japan. Second, no prior study globally has examined how geographic access varies by treatment modality—for example, how access differs for external-beam radiotherapy (EBRT), IMRT or brachytherapy. This is a critical gap because modality availability is unevenly distributed: whereas EBRT capability is widespread, IMRT and brachytherapy are offered by far fewer facilities and are disproportionately concentrated in metropolitan areas. As a result, requiring advanced modalities may impose an additional ‘time tax’ on patients living in rural, mountainous or island regions—an issue of particular concern in Japan’s rapidly aging society.

Recent work has also highlighted the value of geographic information systems (GISs) and travel-time modeling for evaluating RT access and informing service configuration [18]. Building on these methodological advances, the primary objective of this study was to quantify nationwide, time-based geographic access to RT in Japan with explicit stratification by treatment modality (EBRT, IMRT and brachytherapy). By comparing modality-specific travel times against EBRT as a baseline, we aimed to identify geographic areas where requirements for advanced RT impose additional access burdens that are not captured by conventional, non-stratified assessments. Specifically, we (i) quantified national travel-time distributions to EBRT, IMRT and brachytherapy; (ii) estimated the incremental burden incurred when advanced modalities are required and (iii) identified new ≥120-min poor-access areas for each modality. This study provides a comprehensive evidence base for future optimization of RT facility placement, an increasingly important priority in Japan’s super-aging population.

## MATERIALS AND METHODS

### Study design and overview

We conducted a nationwide, cross-sectional geospatial analysis of one-way driving time from residential locations to RT facilities in Japan. Accessibility was evaluated separately for three treatment categories: (a) EBRT using megavoltage photon or electron beams, excluding particle therapy centers; (b) IMRT and (c) brachytherapy. Travel times were estimated from the centroids of all 1-km (third-order) statistical mesh cells to the nearest facility capable of providing each modality. In addition to household-weighted travel-time distributions, two modality-sensitive accessibility measures were derived: modality-specific penalty maps describing incremental minutes required relative to EBRT (truncated at 120 min), and ‘newly ≥120-min’ maps identifying mesh cells that became newly poor-access when IMRT or brachytherapy was required. All travel times represent one-way estimates.

### Population grid and weighting

Residential origins were defined as the centroids of all 176 964 cells of the 1-km third-order statistical mesh covering Japan. For each mesh cell $i$, the number of households was obtained from the 2020 Population Census of Japan (mesh statistics) published by the Statistics Bureau of Japan and was used as the analysis weight in all national and prefectural summaries. The final analytic dataset represented a total of 55 704 949 households nationwide, which served as the denominator for all household-weighted national proportions. Mesh geometries were obtained from the National Land Numerical Information database and harmonized to the EPSG:4326 geographic coordinate system before centroid computation. Mesh cells containing zero households were excluded. All national analyses used household-weighted averages or proportions unless otherwise stated.

### Facility dataset and modality classification

A nationwide list of RT facilities was compiled through manual extraction from publicly available hospital information and institutional sources. Facility names and addresses were cross-checked and verified by hand to ensure completeness and accuracy. For IMRT and brachytherapy, modality capability was determined using the official insurance-designation lists published by the Regional Bureaus of Health and Welfare of the Ministry of Health, Labour and Welfare. Facilities were classified as IMRT-capable or brachytherapy-capable if they were registered as insured medical institutions authorized to claim reimbursement for IMRT or image-guided brachytherapy. Brachytherapy-capable facilities were defined as those authorized under the national insurance system to provide image-guided high-dose-rate (HDR) brachytherapy. Low-dose-rate (LDR) permanent prostate seed implantation was not included, as it is reimbursed under a separate category. These administrative designation lists constitute the authoritative registry of facilities eligible to provide each modality under the national insurance system. After geocoding and verification, the final dataset included 795 EBRT-capable facilities, 419 IMRT-capable facilities and 127 brachytherapy-capable facilities across Japan.

### Road network and routing engine

Driving times were estimated using the Open Source Routing Machine (OSRM) with the car profile and an OpenStreetMap snapshot dated 28 August 2025. Congestion and time-of-day effects were not modeled, yielding free-flow travel-time estimates. Ferry routes were excluded from the routing network, and travel times represent only drivable road connections. Mesh cells accessible solely by ferry were therefore classified as no-route. For each mesh centroid, OSRM was used to compute the shortest one-way driving time to all RT facilities. This matrix-based computation enables efficient evaluation of national-scale origin–destination travel times. For each modality, the minimum feasible travel time to the nearest capable facility was adopted as the travel-time estimate. Mesh cells without a drivable connection were classified as no-route.

### Accessibility metrics

For each modality, household-weighted mean and median travel times were calculated. In addition, we estimated the proportion of all households residing ≥60, ≥90 and ≥ 120 min from the nearest facility capable of providing the relevant modality. The proportion of no-route households was also computed. Unless otherwise stated, all national and prefectural results were weighted by the number of households in each 1-km mesh cell.

### Penalty metric and threshold transitions

Modality-specific incremental burden relative to EBRT was quantified using a 120-min truncated penalty:


$$ {\Delta }_i^m=\max \left\{0,\min \left({T}_i^m,120\right)-\min \left({T}_i^{EBRT},120\right)\right\} $$

Here, *i* indexes each 1-km mesh cell, and *m* denotes the treatment modality (EBRT, IMRT or brachytherapy). ${T}_i^m$ represents the one-way travel time (in minutes) from mesh cell *i* to the nearest facility capable of providing modality *m*, and ${T}_i^{EBRT}$ denotes the corresponding travel time to the nearest EBRT-capable facility.

Newly poor-access cells were identified using:


$$ {N}_{120}^m(i)=1\left\{{T}_i^{EBRT}<120\ and\ {T}_i^m\ge 120\right\} $$

These two measures capture areas where EBRT is locally accessible but advanced-modality capability is substantially farther away, resulting in additional travel burden for IMRT or brachytherapy. The penalty highlights regions where patients would need to travel longer when advanced modalities are required, whereas the threshold-transition indicator identifies communities that newly become ≥120-min poor-access areas only for IMRT or brachytherapy. Reverse transitions were not expected due to the nested structure of modality availability.

## RESULTS

**Fig. 1:**
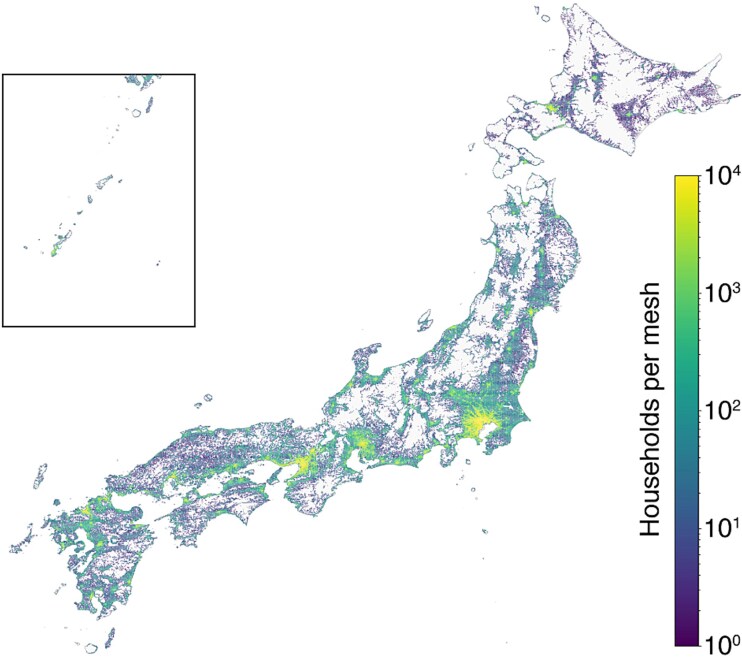
Household distribution by 1-km mesh (log scale). Centroids were used as origins for travel-time computation.

Spatial patterns of residential distribution are shown in Fig. 1, with dense household clusters along major metropolitan corridors and coastal regions, and lower densities in mountainous interiors and remote islands.

**Fig. 2:**
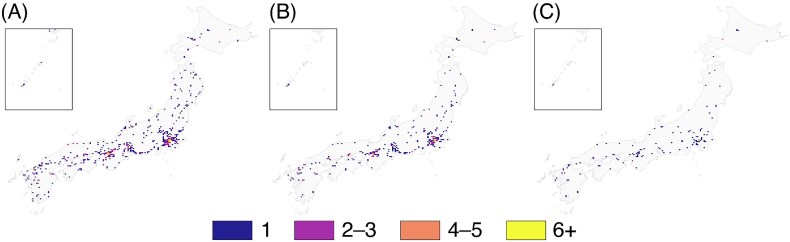
Number of radiotherapy facilities per 10-km mesh: (**A**) EBRT, (**B**) IMRT, (**C**) brachytherapy.

The geographic distribution of RT capability is presented in Fig. 2. EBRT facilities are relatively widespread across Japan, whereas IMRT-capable and brachytherapy-capable facilities are increasingly concentrated in large metropolitan areas.

**Fig. 3:**
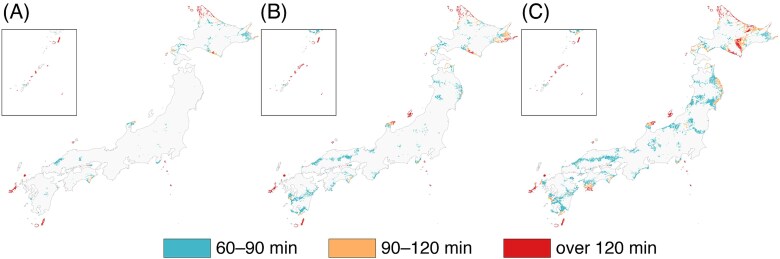
One-way driving-time categories from 1-km meshes to the nearest facility: (**A**) EBRT, (**B**) IMRT, (**C**) brachytherapy.

Household-weighted one-way driving-time categories are mapped in Fig. 3. EBRT access is short for most of the population, with long-travel cells (90–120 min and ≥ 120 min) confined primarily to peninsulas, islands and mountainous belts. When IMRT is required, these long-travel areas expand, with an increase in the proportion of households located ≥60 min from the nearest facility, particularly in Chugoku, Tohoku, Shikoku and Hokkaido. Brachytherapy requirements further enlarge these regions and introduce additional long-travel pockets.

**Fig. 4:**
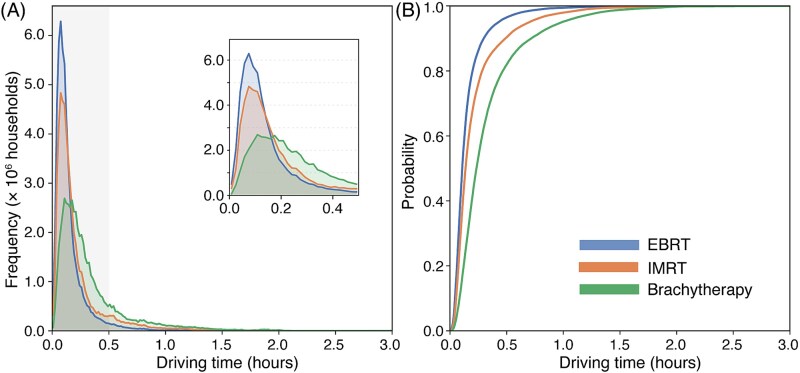
Household-weighted travel-time distributions. (**A**) Histograms with a magnified 0–30-min inset highlighting where most households are concentrated. (**B**) Cumulative distribution functions showing the progressive rightward shift from EBRT to IMRT and brachytherapy.

National travel-time distributions are summarized in Fig. 4 and Table 1. Household-weighted median travel times were 6.48 min for EBRT, 8.26 min for IMRT and 14.06 min for brachytherapy, with corresponding means of 11.00, 17.02 and 23.93 min, respectively. The proportion of all households located ≥60 min from the nearest facility was 0.75% for EBRT, 2.32% for IMRT and 5.09% for brachytherapy. At ≥90 min, the corresponding values were 0.33%, 0.73% and 1.49%; and at ≥120 min, 0.24%, 0.38% and 0.48%. No-route households were rare (0.11–0.14%). Cumulative distribution curves (Fig. 4B) show progressive rightward displacement of the entire distribution with modality escalation, especially for brachytherapy.

**Table 1:** Household-weighted national travel-time metrics by modality

	Weighted median time (min)	Weighted mean time (min)	Households ≥60 min	Households ≥90 min	Households ≥120 min	No-route households
EBRT	6.48	11.00	0.75% (*n* = 418 448)	0.33% (*n* = 184 165)	0.24% (*n* = 132 643)	0.11% (*n* = 62 811)
IMRT	8.26	17.02	2.32% (*n* = 1 290 391)	0.73% (*n* = 408 137)	0.38% (*n* = 210 303)	0.14% (*n* = 75 453)
Brachytherapy	14.06	23.93	5.09% (*n* = 2 836 625)	1.49% (*n* = 827 789)	0.48% (*n* = 269 487)	0.14% (*n* = 75 453)

Proportions are calculated out of a total of 55 704 949 households.

**Fig. 5:**
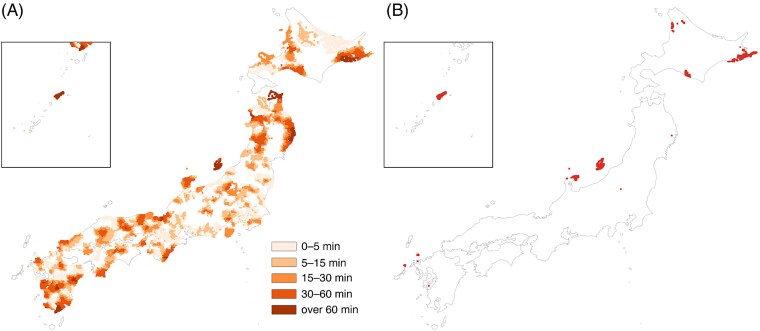
Modality-specific travel-time penalty relative to EBRT for IMRT. (**A**) Additional minutes Δ (0–5, 5–15, 15–30, 30–60, >60 min), truncated at 120 min. (**B**) Meshes newly ≥120 min when IMRT is required (red).

**Fig. 6:**
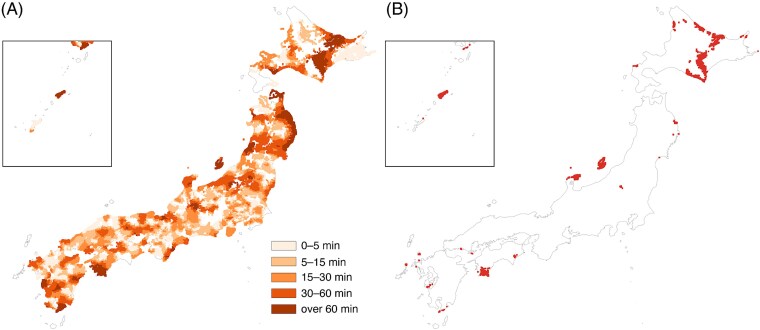
Modality-specific travel-time penalty relative to EBRT for brachytherapy. (**A**) Additional minutes Δ (0–5, 5–15, 15–30, 30–60, >60 min), truncated at 120 min. (**B**) Meshes newly ≥120 min when brachytherapy is required (red).

Modality-specific incremental burdens are mapped in Figs 5 and 6. Additional-minute penalties relative to EBRT (Δ, truncated at 120 min) form geographically coherent clusters in mountainous interiors and island chains, with smaller incremental penalties for IMRT and larger penalties for brachytherapy. Newly poor-access meshes—those that were <120 min for EBRT but become ≥120 min for IMRT or brachytherapy—are sparse for IMRT (Fig. 5B) but more numerous and geographically dispersed for brachytherapy (Fig. 6B).

## DISCUSSION

This study provides, to our knowledge, the first nationwide assessment of geographic access to RT in Japan, and quantified access separately for EBRT, IMRT and brachytherapy. While prior studies from other countries have examined how travel distance affects RT utilization or treatment decisions, none have combined nationwide coverage, high spatial resolution and modality stratification, nor have they evaluated modality-specific geographic inequities at the national scale.

A study from the USA has shown that greater travel distances to RT facilities have been associated with altered treatment patterns, including higher mastectomy rates and lower use of breast-conserving surgery with adjuvant RT among women living farther from treatment centers [13]. Another US registry-based study examined whether travel time influenced treatment decisions for localized prostate cancer and found that most rural patients lived within 30 min of a RT facility and that travel time was not strongly associated with the choice between EBRT and single-visit treatment options, although selected subgroups showed potential interactions [14]. More broadly, a recent systematic review reported mixed and heterogeneous findings, with some studies showing associations between greater travel distance or time and poorer survival, reduced adherence or lower likelihood of receiving guideline-concordant RT, while others found no clear association [15]. Our findings align with these observations but extend them in two important ways. First, we quantify travel-time distributions at nationwide 1-km resolution, capturing fine-grained geographic heterogeneity that is not observable in distance-based regional summaries. Second, we demonstrate modality-specific disparities—particularly for IMRT and brachytherapy—that cannot be detected when RT access is evaluated as a single undifferentiated service.

Although access to EBRT is uniformly short across Japan, with a median travel time of 6.48 min, requiring advanced modalities increased the share of households living ≥120 min from the nearest capable facility. These incremental increases—visible as geographically coherent bands in the penalty maps—represent a ‘time tax’ that may influence treatment decisions or adherence, consistent with patterns described in prior clinical studies [13–15]. Because RT typically requires daily travel over multiple weeks, increases in one-way travel time can translate into cumulative burden, especially for patients with limited mobility or those residing in sparsely populated, mountainous or island regions.

The study also has implications for RT capacity planning. The modality-specific penalty and newly ≥120-min maps identify high-priority regions where upgrading EBRT facilities to IMRT or brachytherapy capability would yield the greatest reduction in travel burden. These spatial patterns are consistent with national structural and utilization surveys demonstrating that advanced modalities in Japan are disproportionately concentrated in larger hospitals and urban prefectures, with substantial inter-prefectural variation in IMRT adoption [1–8]. Identifying areas where EBRT is locally available but higher-level modalities remain geographically distant enables targeted investment strategies—such as upgrading selected regional centers—that can achieve maximal reductions in travel burden with minimal resource expenditure. This form of geographically informed service configuration is consistent with prior work suggesting that geospatial mapping can help inform the siting of RT facilities [19]. Given Japan’s geography, demography and uneven distribution of advanced techniques, adopting similar evidence-based approaches appears warranted. In major metropolitan areas, where many patients rely primarily on public transportation rather than private cars, travel times may differ from the car-based estimates used in this study. Access may be underestimated in settings where traffic congestion substantially prolongs driving time, but it may also be overestimated in areas where rail-based transit offers faster point-to-point travel than driving. Accordingly, actual accessibility in urban regions may deviate in both directions from the estimates reported here.

These considerations are particularly important because Japan is one of the world’s most rapidly aging societies. As older adults constitute a growing share of the population—and as mobility declines, especially in rural areas—the feasibility of completing multi-week RT courses will depend increasingly on geographic proximity to modality-appropriate facilities. Regular nationwide audits using standardized, high-resolution indicators such as those developed in this study may therefore support evidence-based decisions regarding where to maintain, expand or consolidate RT equipment and services in the coming decades. Similar GIS-based simulations have been used to project future changes in medically underserved areas for primary care [20], suggesting that the spatial indicators presented here could support analogous planning for RT.

This study has limitations. Travel times were modeled only for private car travel under free-flow conditions; public transportation, congestion and time-of-day variation were not included. Facility capacity, staffing and case-mix were not accounted for. Geocoding and road-network representations may introduce local uncertainties. The nationwide facility list was compiled through manual extraction from publicly available sources, which may introduce the possibility of omissions, particularly for EBRT facilities that lack centralized registries. Extensive cross-checking was performed to minimize such errors, but completeness cannot be guaranteed. Furthermore, our definition of modality capability was based on facilities formally designated under the national insurance system, representing potential rather than realized access; some designated facilities may not be actively delivering treatment due to staffing limitations or temporary suspension of services. In addition, brachytherapy capability in this study was restricted to image-guided HDR procedures, and facilities performing LDR permanent prostate seed implantation were not included. Our estimates reflect ‘potential access’ based on nearest-capable facility assumptions and may not fully capture patient choice or referral patterns. Therefore, our results should be interpreted as a conservative estimate representing a ‘best-case’ scenario; actual access barriers in clinical practice may be more severe than indicated by these models. Nonetheless, sensitivity analyses suggest that the major findings are robust to these methodological constraints.

In conclusion, this study provides the first comprehensive, modality-specific, time-based evaluation of RT access in Japan. While EBRT travel times are uniformly short nationwide, requirements for IMRT and particularly brachytherapy introduce geographically structured inequities and expand the population living ≥120 min from treatment. In light of evidence linking travel burden to treatment utilization and outcomes, these findings underscore the need for strategic, spatially informed optimization of future RT facility and equipment placement. Given Japan’s aging population and increasing mobility limitations, these findings highlight the need to further optimize the spatial distribution of modality-appropriate RT resources to improve geographic equity.
